# A Parallel Image Denoising Network Based on Nonparametric Attention and Multiscale Feature Fusion

**DOI:** 10.3390/s25020317

**Published:** 2025-01-07

**Authors:** Jing Mao, Lianming Sun, Jie Chen, Shunyuan Yu

**Affiliations:** 1 Graduate School of Environmental Engineering, The University of Kitakyushu, Kitakyushu 808-0135, Japan; 2 Department of Information Systems Engineering, The University of Kitakyushu, Kitakyushu 808-0135, Japan; sun@kitakyu-u.ac.jp; 3 School of Electronic and Information Engineering, Ankang University, Ankang 725000, China; chj526610398@126.com (J.C.); ysywzhm@163.com (S.Y.)

**Keywords:** image denoising, deep learning, nonparametric attention, dilation convolution, residual learning

## Abstract

Convolutional neural networks have achieved excellent results in image denoising; however, there are still some problems: (1) The majority of single-branch models cannot fully exploit the image features and often suffer from the loss of information. (2) Most of the deep CNNs have inadequate edge feature extraction and saturated performance problems. To solve these problems, this paper proposes a two-branch convolutional image denoising network based on nonparametric attention and multiscale feature fusion, aiming to improve the denoising performance while better recovering the image edge and texture information. Firstly, ordinary convolutional layers were used to extract shallow features of noise in the image. Then, a combination of two-branch networks with different and complementary structures was used to extract deep features from the noise information in the image to solve the problem of insufficient feature extraction by the single-branch network model. The upper branch network used densely connected blocks to extract local features of the noise in the image. The lower branch network used multiple dilation convolution residual blocks with different dilation rates to increase the receptive field and extend more contextual information to obtain the global features of the noise in the image. It not only solved the problem of insufficient edge feature extraction but also solved the problem of the saturation of deep CNN performance. In this paper, a nonparametric attention mechanism is introduced in the two-branch feature extraction module, which enabled the network to pay attention to and learn the key information in the feature map, and improved the learning performance of the network. The enhanced features were then processed through the multiscale feature fusion module to obtain multiscale image feature information at different depths to obtain more robust fused features. Finally, the shallow features and deep features were summed using a long jump join and were processed through an ordinary convolutional layer and output to obtain a residual image. In this paper, Set12, BSD68, Set5, CBSD68, and SIDD are used as a test dataset to which different intensities of Gaussian white noise were added for testing and compared with several mainstream denoising methods currently available. The experimental results showed that this paper’s algorithm had better objective indexes on all test sets and outperformed the comparison algorithms. The method in this paper not only achieved a good denoising effect but also effectively retained the edge and texture information of the original image. The proposed method provided a new idea for the study of deep neural networks in the field of image denoising.

## 1. Introduction

In the process of image acquisition and transmission, the original image is often affected by the noise introduced by the system equipment and transmission channel, which leads to the loss of effective information about the image, and then affects the subsequent image analysis and processing, such as image segmentation, target recognition, edge extraction, etc. Accordingly, image denoising has become a classic problem and a popular research topic in the area of vision applications and image processes. Efficient image-denoising algorithms remove the noise while ensuring that the structural information of the processed image is not altered, which helps in other image-processing tasks, and is further used in remote sensing, medical imaging, surveillance, and other fields [1,2].

The current image-denoising algorithms can be classified into two main types, i.e., conventional denoising algorithms and deep learning-based denoising algorithms. The conventional methods mainly use the structural properties of the image itself for denoising, such as denoising algorithms based on the theory of nonlocal self-similarity of the image, denoising algorithms based on sparse representations, and so on. There are ones using filters such as Gaussian filtering methods [3,4], bilateral filtering methods [5,6], and median filtering methods [7,8]. Nonlocal self-similarity algorithms take advantage of the fact that the image blocks in a natural image are similar to each other, and search for image blocks similar to the image block centered on the current pixel in the whole image, and process their similar blocks. The representative ones are nonlocal means (NLMs) [9,10,11], three-dimensional block-matched filtering (BM3D) [12], and the weighted nuclear paradigm minimization (WNNM) [13]. Classical sparse representation-based denoising methods include the dictionary learning algorithm (KSVD) [14], and nonlocal centralized sparse representation (NCSR) [15,16]. However, such methods need to find the a priori information of the image first, and then use optimization algorithms to solve the model iteratively. Therefore, the complex optimization process of traditional denoising methods takes a lot of time and computational cost and also requires manual parameter adjustment, which is not very generalizable. Traditional methods are also prone to image blurring and detail loss problems.

With the enhanced performance and computational power of various types of computers, researchers have gradually introduced deep learning methods into the field of image processing. Deep learning has been applied broadly in the area of computer vision [17,18,19,20,21,22]. The main ideas of the deep learning denoising algorithm are to use a large number of noisy and clean image pairs and to perform deep neural network denoising of these training data using end-to-end learning with excellent performance. Schmidt and Roth proposed a cascade of shrinkage fields (CSF) [23] approach to unify random field-based models and unfold semi-quadratic optimization algorithms into a single learning framework. Chen [24] et al. proposed a trainable nonlinear reaction–diffusion (TNRD) model. Burger [25] et al. implemented image denoising using a multilayer perceptron (MLP) approach. ZHANG et al. [26] raised a deep denoising convolutional neural network DnCNN, which for the first time applied batch normalization [27] and residual learning [28] to the field of image denoising, and was able to handle uniform Gaussian noise effectively. Subsequently, ZHANG et al. [29] proposed an FFDNET method for image denoising, which took the noise level and the noisy image as joint inputs and trained a model to process the noisy image under different noise levels. To further optimize the denoising performance of the neural network, Tian [30] offered an enhanced convolutional network, ECNDNet, by combining the dilation convolution with ordinary convolution, which further improved the sensory field of the network. The author of [31] introduced residual optimization based on a convolutional neural network, which addressed the progressive disappearance of the gradient during the propagation of convolutional neural networks when the number of layers was greater. The author of [32] set a baseline depth denoising initially by training a flexible and efficient CNN denoiser, which was inserted as a module into an iterative HQS-based algorithm that could solve various image restoration problems. The author of [33] proposed a robust deformation denoising CNN that could exploit morphable learned kernels and stamped convolutional architectures to extract more typical noise features. Study [34] was a mixture of denoising models based on a network of transformer encoders and convolutional decoders that achieved state-of-the-art denoising performance on real images at relatively low computational cost. The author of [35] designed a dual network with a sparse mechanism that extracts complementary features to recover clear images that could act on real noisy images.

Although the above deep learning-based denoising algorithms have produced good results, there are still problems. The edge and texture information of the image is very important for the recovery of the image, but the denoising network treats all the acquired information in the network equally and does not focus on the edge and texture information of the input image, which results in the poor recovery of the denoised image in the edge region. Therefore, how to extract the edge as well as texture features of the image from the limited features is the difficulty of the subsequent denoising network. To address the above problems, Hu et al. [36] proposed a channel attention mechanism to learn the correlation between channels. Woo et al. [37] proposed CBAM to better learn the correlation between feature maps from channels and spatial locations. These two attention mechanisms generated weights through global pooling operations and convolution. Yang [38] proposed SimAM (a simple parameter-free attention module) to learn the correlation between channels and spatial correlations at each position of the feature map without the need for parameters, using statistical laws. In addition to single-layer convolutional neural networks, BRDnet [39] with a two-layer neural network structure has also been proposed, which increased the width of the network by combining two networks to obtain more features and improved the training speed and training effectiveness by applying batch renormalization, residual learning, and dilation convolution simultaneously. Z. Cai et al. [40] proposed a two-stage image denoising model in which the input image was first processed with a specialized denoiser, and then the resulting intermediate denoised image was passed to a kernel prediction network that estimated the denoising kernel for each pixel. The robustness of the method to noise parameters superseded comparable blind denoisers while approaching state-of-the-art denoising quality for camera sensor noise.

Based on the previous research, this paper presents a parallel denoising network with nonparametric attention and multiscale feature fusion (NAMFPDNet). The main work is as follows:

(1) Aiming at the recovered image with blurred edge information and unclear image texture, a dual-branch image denoising network (NAMFPDNet) based on the residual denoising network is proposed based on the nonparticipant attention mechanism and multiscale feature fusion.
(2) A dual-branch deep feature extraction module was designed, in which the upper branch adopted the densely connected block to extract the local features of image noise, and the lower branch combined the ordinary convolution with the dilated convolution to form the residual block, which extracted the global information of image noise and strengthened the feature extraction capability of the network. Compared with the single-branch network structure, the dual-branch network not only solved the problem of insufficient feature extraction by the single-branch network model but also solved the problem of the saturation of the deep CNN performance.
(3) We used SimAm, a parameter-free attention mechanism. A parameter-free attention module was designed to focus on critical regions in important channels in the feature map from both spatial and channel aspects so that the network could recover clear edges as well as texture details.
(4) We designed a multiscale feature fusion module that deeply fuses global and local features using three convolutional layers of different scale sizes. Compared with the traditional single-scale convolution operation, the multiscale feature fusion method could better recover the image contour information and texture information.

## 2. Theory and Methodology

**Figure 1:**
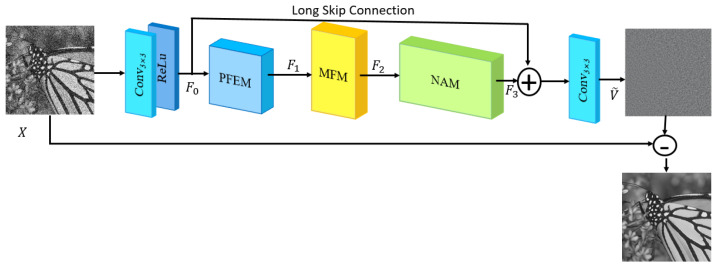
The framework of the NAMFPDNet.

The paper designs a parallel image denoising network based on nonparametric attention and multiscale feature fusion (NAMFPDNet). The network continued the idea of DnCNN [24] by using residual learning combined with batch normalization. The denoising network structure is shown in Figure 1. The input of this network was the noisy image X and the output was the residual image $\tilde{V}$ learned by the NAMFPDNet. The purpose of this algorithm was to learn the residual $\tilde{V}$ that approximated the noise V, i.e., $\tilde{V}=NAMFPDNet(X)\approx V$, and then remove the residual $\tilde{V}$ from the noisy image X.

The entire network first applied a Conv_3×3_ + ReLU to perform an initial sampling operation on the image X. The size of the convolution kernel was 3 × 3. There were 1 input channel and 64 output channels. The initial feature extraction module was used to extract the initial features $F_{0}.$ Then, $F_{0}$ was passed through the parallel feature extraction module (PFEM), which adopted the upper branch network and the lower branch network in parallel to obtain the depth features of an image, and the features were spliced to obtain the deep features $F_{1}.$ Then, $F_{1}$ was passed through a multiscale feature fusion module (MFM) for feature fusion to obtain the fused feature $F_{2}.$ The fused feature $F_{2}$ was passed through the nonparametric attention module (NAM), which focused on the critical regions in the important channels in the feature map both spatially and channel-wise, so that the network could recover clear edges as well as texture details to obtain the enhanced feature $F_{3}$.

The shallow feature $F_{0}$ and the enhanced deep feature $F_{3}$ were merged using a long skip connection and then passed to the residual reconstruction module. The residual reconstruction module applied only one layer of convolution to reconstruct the noise residuals, the size of the convolution kernel was 3 × 3, the number of input channels was 64, and the number of output channels was 1. The noise residual map $\tilde{V}=NAMFPDNet(X)$ learned by the network was shown as shown in Equation (1). Long jump connections integrated shallow and deep feature information in the network, which was beneficial to stabilize the training of the network and improve the denoising performance. Finally, the original image was used to subtract $\tilde{V}$ to obtain the denoised image.

(1) $$NAMFPDNet(X)=Conv_{3\times 3}(F_{0}+F_{3})$$

### 2.1. Parallel Feature Extraction Module

**Figure 2:**
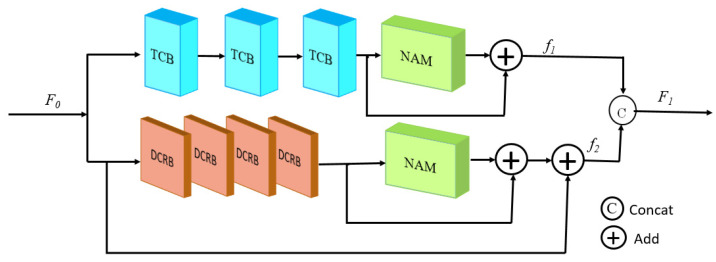
Structure of PFEM.

The parallel feature extraction module (PFEM) employed a network structure in which the upper branch network and the lower branch network were connected in parallel. This is shown in Figure 2.

The upper branch network in this paper used a similar connectivity approach as DenseNet, which mainly consisted of three tightly connected blocks (TCBs) connected in series to extract the local features of the noisy image. This local feature was processed by the later nonparametric attention mechanism (NAM) module and using the local residual connections. NAM learned the correlation of each position from the spatial and channel positions for the extracted features, and thus adaptively changed the weight of each position, which was multiplied with the extracted features, to achieve the focus on the important features in the local specialization and suppression of the invalid features. The output of the upper branch network was $f_{1}.$

**Figure 3:**
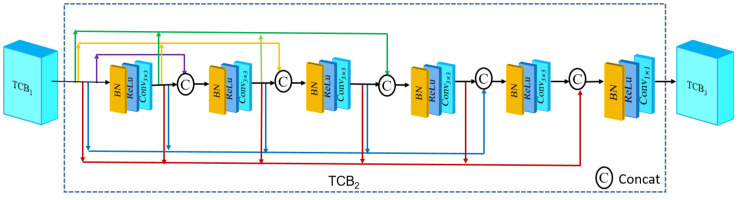
TCB structure.

The structure of TCB is shown in Figure 3, and the input of the normalization layer in TCB came from the output of all the previous convolutional layers. This dense connection not only solved the gradient vanishing problem, but also brought powerful feature extraction capability and enhanced feature propagation. The TCB consisted of a total of five convolutional layers with a convolutional kernel size of 3 × 3, and the parameter settings of the convolutional layers are shown in Table 1. A convolutional layer of a 1 × 1 size was used to reduce the number of channels at the end of the TCB, and the number of channels of the output feature map was 64, thus effectively reducing the computation.

The down-branch network in this paper used four dilated convolutional residual blocks (DCRBs) in series, which were then subjected to NAM for feature enhancement. The purpose of the design of the lower branch network was to compensate for the damage to the image information structure and the loss of noise information in the first branch through different structures. The output of the down-branch network was $f_{2}.$

**Figure 4:**
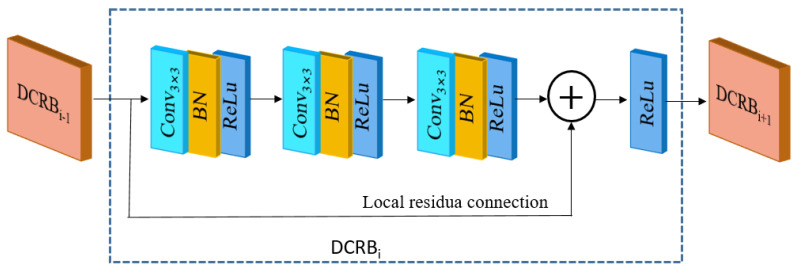
DCRB structure.

The structure of the DCRB is shown in Figure 4. The DCRB mainly consisted of a series of dilated convolutions with dilatation rates of 1, 2, and 3, respectively. The size of the convolution kernel was 3 × 3, with 64 convolution kernel numbers. The network specific parameter settings are shown in Table 2.

Combining the dilation convolution with ordinary convolution, forming a sparse structure, expanded the sensory field of the network without additional learning parameters, which solved the problem of saturating the network feature extraction caused by using a single-sized convolutional kernel for deep networks, and effectively improved the performance of denoising networks. Different dilation rates prevented the lattice effect brought about by a single dilation rate. Local residuals were also added inside the DCRB to further enhance the feature extraction capability of the module, thus improving the model performance.

### 2.2. The Nonparametric Attention Module

Yang [38] proposed a simple attention module (SimAm) by statistical laws, and SimAm proposed a 3d attention module based on human visual neurons focusing on both spatial as well as channel attention. Specifically, an energy function was optimized based on many neuroscience theories to find the importance of each neuron. By designing the linear separability between the target neuron and other neurons in the same channel, it was determined whether the neuron should be attended to or not. By deriving a closed-form solution of the energy function, the minimum energy of the neuron was obtained as shown in Equation (2).

(2) $$e_{t}^{*}=\frac{4({\hat{\delta }}^{2}+\lambda )+0.5}{{\left(t-\hat{\mu }\right)}^{2}}$$

where $\hat{\mu }=\frac{1}{M-1}\sum_{i=1}^{M-1}x_{i}$; ${\hat{\delta }}^{2}=\frac{1}{M-1}\sum_{i=1}^{M-1}{\left(x_{i}-\hat{\mu }\right)}^{2}$.

X indicates the input feature map, $X\epsilon R^{H*W*C}$, and M=H ∗ W indicates the number of neurons in the same channel. $\hat{\mu }$ denotes the mean of all neurons within the same channel. ${\hat{\delta }}^{2}$ represents the energy variance of the neurons within the same channel. λ was taken as 1e−4. Lower energy meant that the neuron was more deserving of attention than other neurons. Thus, the importance of a neuron can be obtained by $1/e_{t}^{*}$.

Since attention was achieved by weighting, the formula for SimAm is shown in Equation (3),

(3) $$\tilde{X}=Sigmoid(1/e_{t}^{*})⊙X$$

Based on SimAm theory, the specific steps for the implementation of the nonparametric attention module (NAM) designed in this paper were as follows:

Input: X represents the input feature map, $X\epsilon R^{H*W*C};$

Output: enhanced feature map $\tilde{X}$.

Step 1: Calculate the mean value of X over the channel dimension. This meant squeezing the feature map along the spatial direction to find the mean value on each H ∗ W.

Step 2: Calculate the square of the mean error for each position in the same channel, obtaining $X^{\prime \;}\mathrm{and}\;X^{\prime }\epsilon R^{H*W*C}$.

Step 3: Compute the variance of X over the channel dimensions. That meant that X′ was summed over each H ∗ W dimension and divided by n, where n=H ∗ W−1. Then, obtain the result *t* $and\;t\epsilon {R\;}^{1*1*C}$, as the channel attention information.

Step 4: Calculate the amount of energy per pixel by using $(4\;*\;\left(t+\lambda \right)+0.5)/X\prime$.

Step 5: Enhance the feature map using the sigmoid function. This implied that the $\tilde{X}$ obtained was calculated using Equation (3) as the augmented feature map.

**Figure 5:**
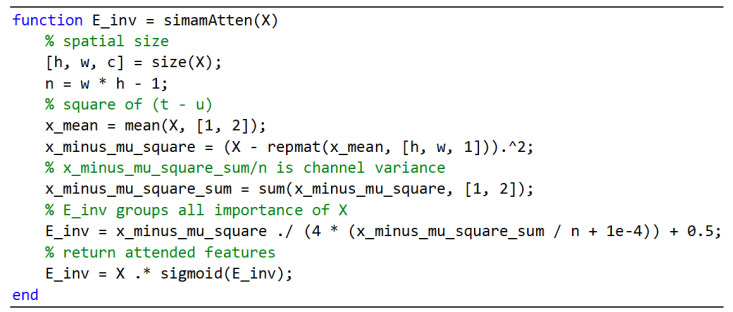
An implementation of simamAtten.

To implement NAM, a custom neural network layer, named NAM, inherited from the nnet.layer.Layer base class was defined. In the forward propagation function of this class, the NAM was implemented. Therefore, the forward propagation method for this layer based on the above steps is shown in Figure 5 below.

Compared with the existing channel attention mechanism SE and mixed attention mechanism CBAM, although the SE and CBAM can greatly improve the accuracy of the network, the network would generate more parameters because its implementation depends on the Full Connectivity Layer and Pooling Layers for weight allocation. For SimAm, on the other hand, it can provide neural networks with three-dimensional attention weights without adding any network parameters, as shown in Table 3. This feature enabled SimAm to greatly reduce the complexity and computational cost of the model while maintaining high performance.

### 2.3. Multiscale Feature Fusion Module

**Figure 6:**
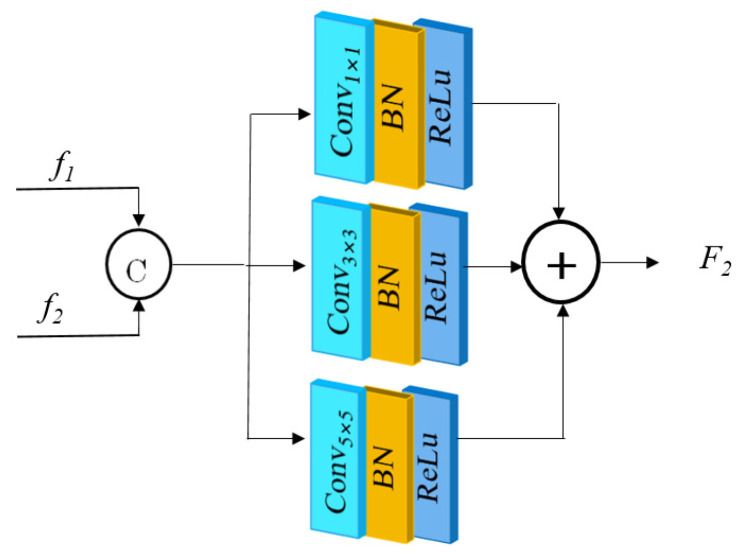
Multiscale feature fusion module.

This paper uses multi-feature fusion to extract image features at different scales. The structure of the multiscale feature fusion block (MFM) is shown in Figure 6. ${\;f}_{1}$ and $f_{2}$ indicate the feature maps’ output from the upper and lower branch networks, respectively. The feature maps of the two branches were first feature-concatenated, and then feature extraction was carried out by parallel convolutional layers of convolutional kernel sizes 1 × 1, 3 × 3, and 5 × 5, respectively, with the number of convolutional kernels being 64. The extracted features were finally summed and fused. As compared to traditional single-scale convolutional operations, the multiscale feature fusion method can better recover the image contour information and texture information.

## 3. Experimental Results and Analyses

### 3.1. Experimental Setting

The training set images were randomly rotated to obtain the enhanced image, and then the enhanced image was cropped into small image blocks of size 40 × 40 pixels. Subsequently, Gaussian white noise was added to the image blocks to generate noise-containing images, to test the effect of noise intensity on network performance. The test set uses Set12, BSD68, Set5, CBSD68, SIDD, and DND image datasets.

The Adam optimizer was employed to train. The initial learning rate was 1e−3, β1=0.9, β2=0.999, and $\varepsilon =10^{-8}$. This paper adopts the learning rate decay rule, and the decay period was 50 epochs. The learning rate decay product factor gamma = 0.1. The training was terminated after 50 epochs. The batch size was set to 128.

### 3.2. Loss Function and Evaluation Metrics

(1) Loss function

For training the models in this paper, the mean square deviation function was used as a loss function to train the network parameters, the mathematical expression of which is given in Equation (4).

(4) $$Loss(\theta )=\frac{1}{2N}\sum_{i=1}^{N}{\left\|NAMFPDNet(Xi;\theta )-(Xi-Ii)\right\|}^{2}$$

where N denotes the number of training samples, θ denotes the parameters learned by the NAMFPDNet, and $\left(X_{i},\;I_{i}\right)$ denotes the corresponding (noisy, clear) image.

(2) Evaluation metrics

In this paper, PSNR and SSIM are used to evaluate the quality of denoised images, as shown in Equations (5) and (6) [41,42].

(5) $$PSNR=10\times \log_{10}\left[\frac{255}{\frac{1}{H\times W}\sum_{i=1}^{H}\sum_{j=1}^{W}{\left[I(i,j)-\tilde{I}(i,j)\right]}^{2}}\right]$$

(6) $$SSIM=\frac{\left(2\mu_{1}\mu_{2}+C_{1})(2\sigma_{1,2}+C_{2}\right)}{\left({\mu_{1}}^{2}+{\mu_{2}}^{2}+C_{1})({\sigma_{1}}^{2}+{\sigma_{2}}^{2}+C_{2}\right)}$$

### 3.3. Comparison Experiment of Denoising Performance

#### 3.3.1. Gray Image Denoising

To prove the denoising performance of the network in the paper, for greyscale images, this paper compares seven more advanced image denoising methods, including BM3D, WNNM, EPLL, MLP, DnCNN-S, DnCNN-B, and FFDNet, on the Set12 dataset and BSD68 dataset.

Table 4 and Table 5, respectively, show the average values of PSNR and SSIM for different denoising methods on the test sets Set12 and BSD68 for three noise levels of 15, 25, and 50, where the best-performing value was given a bold font. It was evident that the average value of PSNR and SSIM for the network in this paper was higher than the other methods. Take Set12, for example; at a noise level of 50, the average PSNR of this paper was 0.83 dB, 0.50 dB, and 1.21 dB higher than the average of BM3D, WNNM, and EPLL, respectively, and 0.37 dB, 0.35 dB, and 0.22 dB higher than the average PSNR of DnCNN-S, DnCNN-B, and FFDNet, respectively. Combined results in Table 4 and Table 5 indicated that this paper’s method can obtain a better PSNR average and SSIM average than other methods in the overall comparison, and this method possessed better denoising performance while maintaining the detailed features of the image in a better way.

**Figure 7:**
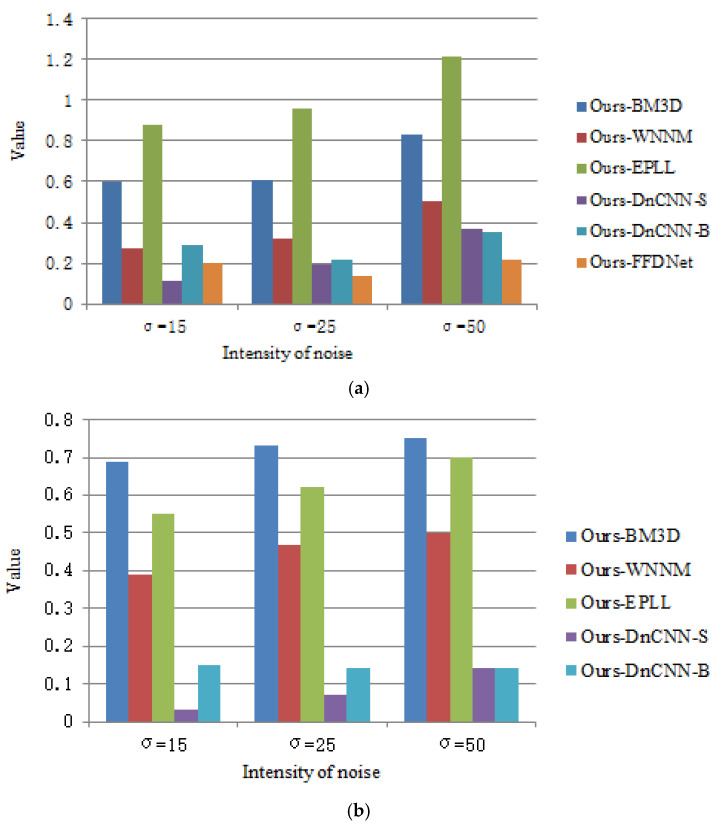
Results of average PSNR of the proposed method minus average PSNR (dB) of other algorithms (on Set12 and BSD68). (**a**) Set12; (**b**) BSD68.

To more intuitively compare the difference between the proposed method and other methods on the Set12 and BSD68 test set, we used average PSNR. Figure 6 shows the bar chart of the difference between BM3D, WNNM, EPLL, MLP, DnCNN-S, DnCNN-B, FFDNet, and the proposed method on Set12 and BSD68. As can be seen from Figure 7, as the noise intensity increased, the difference between the proposed method and other methods also increased, indicating that the proposed method had a better denoising ability for high-intensity noise compared with other methods.

To better compare the image denoising effect of different methods, the values of PSNR and SSIM after denoising by different methods are given in Table 6, Table 7, Table 8, and Table 9 for eight images on Set12, respectively. It can be seen that the PSNR values of this paper’s method were better than the other methods for most of the test images at Gaussian noise levels of 15 and 25. The SSIM values of this paper’s method at all noise intensities were located in first place or second place, indicating that this paper’s denoising method better restored the image structure. In summary, the method in this paper achieved better results in two objective metrics, indicating that the method had better noise.

**Figure 8:**
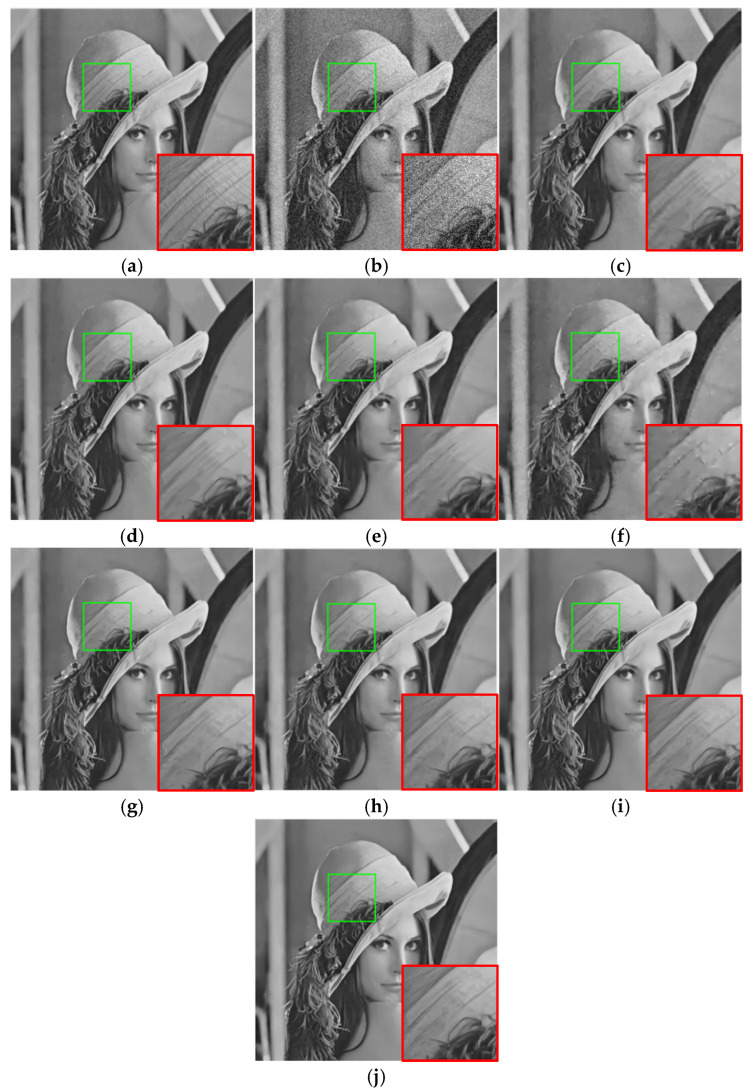
Lena image denoising results (δ=25). (**a**) Original image; (**b**) noise image/20.26 dB; (**c**) BM3D/32.07 dB; (**d**) WNNM/32.24 dB; (**e**) MLP/32.25 dB; (**f**) EPLL/31.73 dB; (**g**) DNCNN-S/32.44 dB; (**h**) DNCNN-B/32.42 dB; (**i**) FFDNet/32.57 dB; (**j**) ours/32.74 dB.

**Figure 9:**
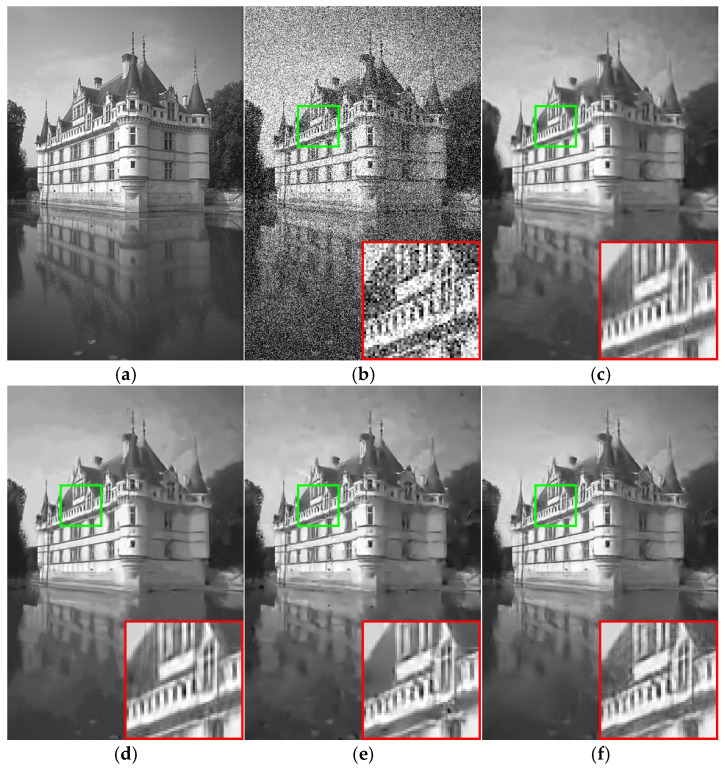

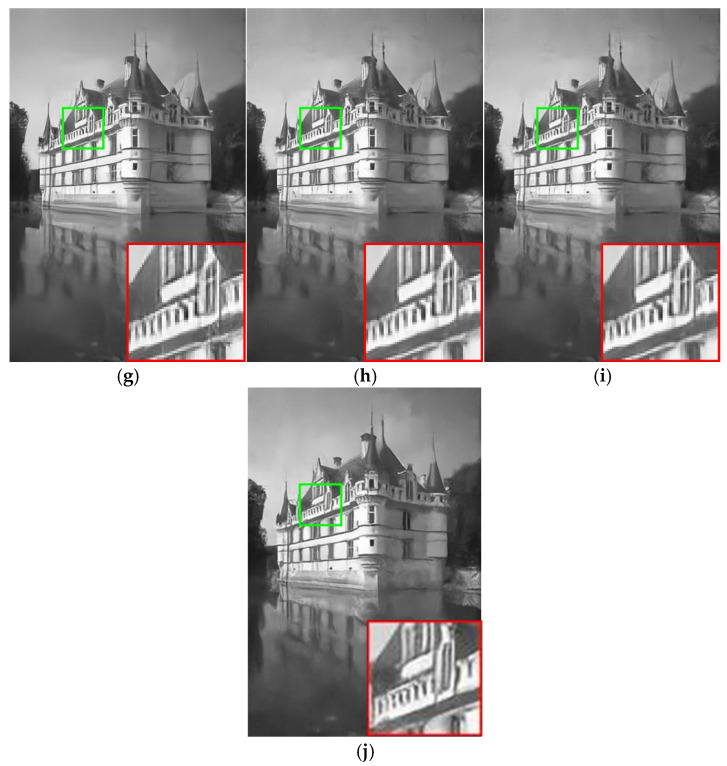
Building image denoising results (δ=50). (**a**) Original image; (**b**) noise image/14.78 dB; (**c**) BM3D/26.21 dB; (**d**) WNNM/26.51 dB; (**e**) EPLL/26.36 dB; (**f**) MLP/26.54 dB; (**g**) DnCNN-S/26.89 dB; (**h**) DnCNN-B/26.92 dB; (**i**) FFDNet/26.93 dB; (**j**) ours/26.97 dB.

To show the efficiency of the proposed algorithm more clearly, an image was randomly selected from each of the two test datasets as the subjective evaluation result of the image denoising effect. The subjective results of the different algorithms are shown in Figure 8 and Figure 9 below, in which the magnified details of the local regions of the images are shown in the bottom right corner of each image. It can be seen that the edge of the image after denoising by most of the comparison algorithms was relatively blurred, such as BM3D, WNNM, and MLP algorithms. The EPLL method not only lost many details in the image but also still left a small amount of noise, while the image appeared as artifacts. Therefore, the algorithm in this paper was comparable to the visual denoising effect of DnCNN and FFDNet algorithms. This paper’s algorithm was more capable of restoring the texture details of the original image, and this contrast effect was more obvious in the case of that more seriously polluted by noise.

#### 3.3.2. Color Image Denoising

In this paper, Set5 and CBSD68 are used as color image datasets to verify the effectiveness of the proposed algorithm on color image denoising. The selected comparison algorithms included CBM3D [10], DNCNN-C [24], and FFDNet [27]. The experimental results are shown in Table 10 below. It can be seen from the table that the proposed algorithm had a better PSNR value than other algorithms.

**Figure 10:**
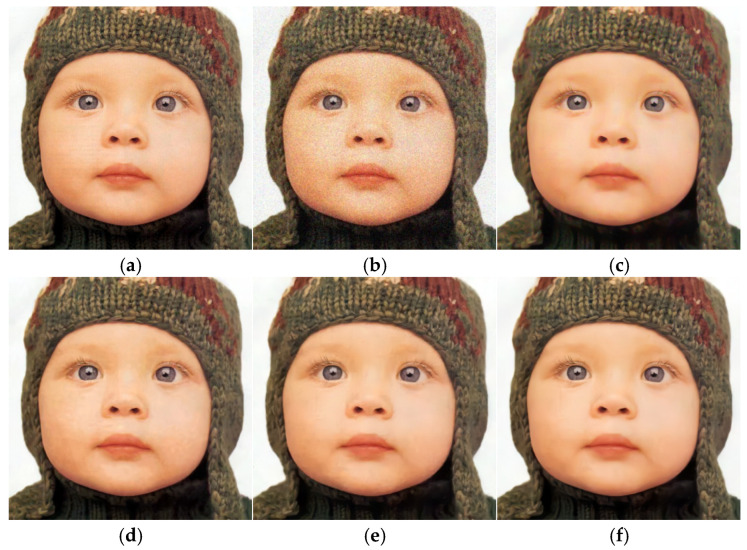
Denoising effect of different algorithms from Set5 (δ=25). (**a**) Original image; (**b**) noise image/20.8186 dB; (**c**) CBM3D/33.1082 dB; (**d**) DNCNN-C/33.1754 dB; (**e**) FFDNet/33.3205 dB; (**f**) ours/33.4017 dB.

**Figure 11:**
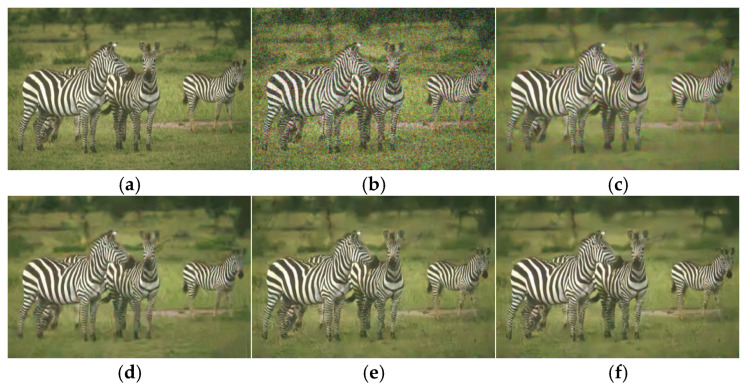
Denoising effect of different algorithms from CBSD68 (δ=50). (**a**) Original image; (**b**) noise image/14.6714 dB; (**c**) CBM3D/26.4053 dB; (**d**) DNCNN-C/27.3557 dB; (**e**) FFDNet/27.2981 dB; (**f**) ours/27.3931 dB.

An image was selected from Set5 and CBSD68 datasets, respectively, for experiments, and the denoising results of the proposed algorithm and other algorithms were presented. As can be seen from Figure 10 and Figure 11, the denoising results of the proposed algorithm were clearer and the details of the image were preserved.

#### 3.3.3. Real Image Denoising

For real image denoising, two real noisy image datasets, SIDD [43] and DND [44], were tested using CBM3D, DnCNN, FFDNet, CBDNet [45], and the algorithm in this paper. The average PSNR and SSIM values of different denoising methods are shown in Table 11. It can be seen that the average PSNR and SSIM of this paper’s algorithm were the best, showing better denoising performance.

**Figure 12:**
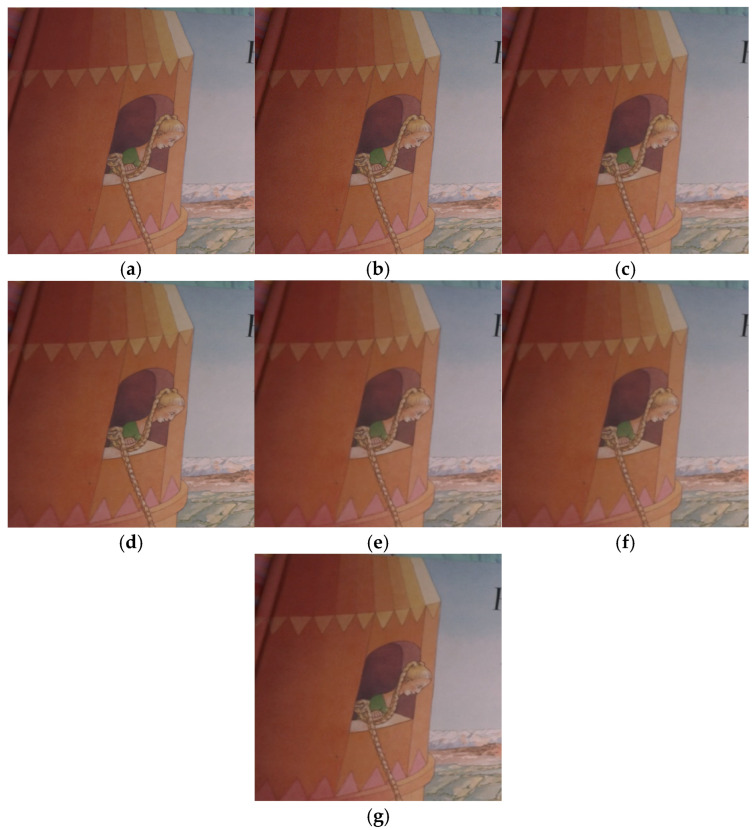
SIDD image denoising results with different algorithms. (**a**) Noise image; (**b**) original image; (**c**) BM3D (35.64 dB); (**d**) DnCNN-C (40.67 dB); (**e**) FFDNet (40.96 dB); (**f**) CBDNet (38.75 dB); (**g**) ours (41.36 dB).

From the SIDD dataset, an image was selected for denoising experiments and the denoising results of this paper’s algorithm and other algorithms are shown. As can be seen in Figure 12, the denoising results of this paper’s algorithm performed well in preserving the detailed features of the denoised image, also compared to CBM3D, DnCNN, and CBDNet.

### 3.4. Ablation Experiments

**Figure 13:**
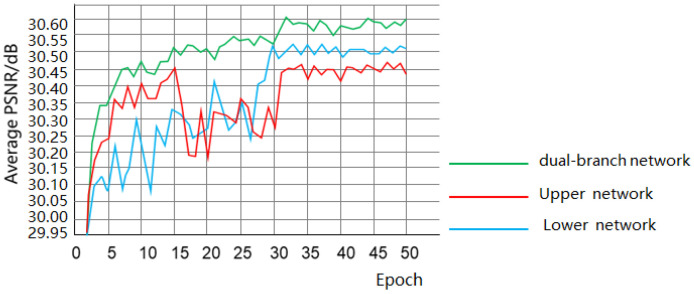
Average PSNR of each sub-network with a dual-branch network.

To test the effectiveness of this paper’s upper and lower branching networks for image Gaussian denoising, the upper and lower branching networks were, respectively, subjected to Gaussian image denoising on the Set12 grayscale map dataset, and the obtained average PSNR value trend (σ = 25) is shown in Figure 13. It can be seen that although the separate up-branch or down-branch networks showed well in the denoising, the denoising performance of the dual-branch network was greatly improved. It proved that the two structures can complement each other to better extract the features of the image and achieve better denoising results.

**Figure 14:**
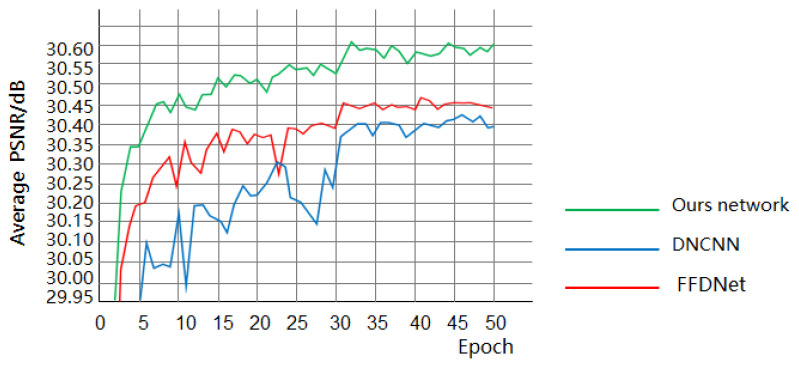
Average PSNR of this paper’s network with DnCNN and FFDNet.

The network in this paper and DnCNN as well as FFDNet were subjected to Gaussian image denoising on the Set12, and the average PSNR value (σ = 25) was obtained as shown in Figure 14. It can be seen that the highest PSNR value of the model in this paper was much higher than that of DnCNN and FFDNet, and its convergence speed was also much faster than that of DnCNN and FFDNet. It fully proved that the network in this paper was advanced in Gaussian image denoising.

**Figure 15:**
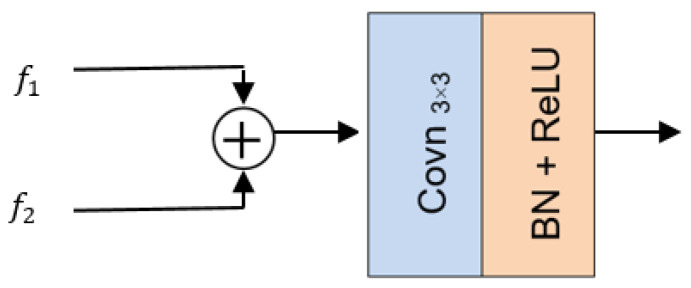
Feature direct fusion method.

To verify the effectiveness of each module in the network, ablation experiments are conducted in this paper and Set12 was chosen for the test set. (1) The network without the nonparametric attention module (NAM) as well as the multiscale feature fusion module (MFM) was used as the baseline network (BL). (2) Adding a nonparametric attention module (NAM) to BL was called baseline with NAM (BL + NAM). (3) The channel attention mechanism was introduced in the baseline network (BL + SE) to be compared with the BL + NAM. (4) We verified the effectiveness of the multiscale feature fusion module. Two methods, the ordinary direct feature fusion method (BL + NAM + direct) and the multiscale feature fusion method (BL + NAM + MFM), were used for comparison, respectively. The ordinary direct feature fusion method is shown in Figure 15 below, and the multiscale feature fusion method is shown in Figure 6.

As can be seen from Table 12 and Table 13, the PSNR and SSIM values of the baseline network were improved after introducing the NAM and the MFM module into the baseline network, indicating that both the NAM and the MFM can effectively improve the denoising performance of the network. The BL + NAM + MFM network had higher PSNR and SSIM values than the BL + NAM + direct network, which confirmed the superiority of the MFM module. The BL + NAM performed better than the BL + SE. As the noise intensity increased, the contribution of each module to the baseline network showed a growing trend, which proved that the network in this paper had better high noise removal capability.

## 4. Conclusions

With the improvement of computer arithmetic power, deep learning has achieved many results in the field of computer vision. To address the problems of a denoised image edge and unclear texture in previous image denoising algorithms based on deep learning, a dual-branch image denoising network based on nonparametric attention and multiscale feature fusion was proposed. The method uses a two-branch network structure for feature extraction to make up for the shortcomings of the single-branch network. Meanwhile, nonparametric attention was introduced into the feature extraction module to focus on the important features spatially and channel-wise to learn and extract the key information effectively. A new multiscale feature fusion approach was also proposed to better fuse local features with global features. Through the analysis of the experimental results, such as objective evaluation and subjective evaluation, the method in this paper had a certain degree of improvement both in terms of indicators and vision and could obtain clearer denoised images and retain more edge detail information. In the future, the study of denoising networks applied on hyperspectral images should be continued, to further optimize the network structure.

## Figures and Tables

**Table 1:** TCB parameter settings.

Network Layer	Kernel Size	Stride	Padding	Output Channels
Conv_3×3_	3 × 3	1	0	64
Conv_1×1_	1 × 1	1	0	64

**Table 2:** DCRB parameter settings.

Network Layer	Kernel Size	Stride	Dilation Factor	Output Channels
The first Conv_3×3_	3 × 3	1	1	64
The second Conv_3×3_	3 × 3	1	2	64
The third Conv_3×3_	3 × 3	1	3	64

**Table 3:** Comparison of parameters of different attention mechanisms.

Attention Models	Parameters	Remarks
SE	$2C^{2}/r$	C: number of current feature channels r: reduction ratio k: number of convolutional layer filters
CBAM	2C2r+2k2	
NAM	0	

**Table 4:** Average PSNR (dB) values of different methods on Set12 and BSD68.

Dataset	σ	BM3D	WNNM	EPLL	DnCNN-S	DnCNN-B	FFDNet	Ours
Set12	15	32.37	32.70	32.09	32.86	32.68	32.77	**32.97**
	25	29.97	30.26	29.62	30.39	30.36	30.44	**30.58**
	50	26.72	27.05	26.34	27.18	27.20	27.33	**27.55**
BSD68	15	31.07	31.37	31.21	31.73	31.61	31.63	**31.76**
	25	28.57	28.83	28.68	29.23	29.16	29.19	**29.34**
	50	25.62	25.87	25.67	26.23	26.23	26.29	**26.37**

The bold one in the table is the best indicator.

**Table 5:** Average SSIM values of different methods on Set12 and BSD68.

Dataset	σ	WNNM	BM3D	DnCNN-S	DnCNN-B	FFDNet	Ours
Set12	15	0.8869	0.8989	0.9027	0.9001	0.9018	**0.9057**
	25	0.8323	0.8553	0.8618	0.8602	0.8628	**0.8668**
	50	0.7282	0.7679	0.7827	0.7828	0.7902	**0.7953**
BSD68	15	0.9094	0.8741	0.8906	0.8866	0.8901	**0.8933**
	25	0.8157	0.8025	0.8278	0.8242	0.8289	**0.8341**
	50	0.7029	0.6744	0.7189	0.7164	0.7242	**0.7301**

The bold one in the table is the best indicator.

**Table 6:** PSNR (dB) values for different denoising methods on Set12 (δ=15).

Method	C.man	Pepper	Molnar.	Airplane	Parrot	Lena	Barbara	Boat
BM3D	31.91	32.69	31.85	31.07	31.37	34.26	33.10	32.13
WNNM	32.17	32.97	32.71	31.39	31.62	34.27	**33.60**	32.27
EPLL	31.85	32.64	32.10	31.19	31.42	33.92	31.38	31.93
DnCNN-S	**32.61**	33.30	33.09	31.70	31.83	34.62	32.64	32.42
DnCNN-B	32.10	33.15	32.94	31.56	31.63	34.56	32.09	32.35
FFDNet	32.43	33.25	32.66	31.57	31.81	34.62	32.54	32.38
**OURS**	32.57	**33.42**	**33.27**	**31.92**	**31.96**	**34.72**	32.89	**33.31**

The bold one in the table is the best indicator.

**Table 7:** PSNR (dB) values for different denoising methods on Set12 (δ=25).

Method	C.man	Pepper	Molnar.	Airplane	Parrot	Lena	Barbara	Boat
BM3D	29.45	30.16	29.25	28.42	28.93	32.07	30.71	29.90
WNNM	29.64	30.42	29.84	28.69	29.15	32.24	**31.24**	30.03
MLP	29.61	30.30	29.61	28.82	29.25	32.25	29.54	29.97
EPLL	29.26	30.17	29.39	28.61	28.95	31.73	28.61	29.74
DnCNN-S	30.18	30.87	**30.28**	29.13	29.43	32.44	30.00	30.18
DnCNN-B	29.94	30.84	30.25	29.09	29.35	32.42	29.69	30.20
FFDNet	30.10	30.93	30.08	29.04	29.44	32.57	30.01	30.25
Ours	**30.37**	**31.13**	30.25	**29.29**	**29.59**	**32.74**	30.81	**30.45**

The bold one in the table is the best indicator.

**Table 8:** SSIM values for different denoising methods on Set12 (δ=15).

Method	C.man	Pepper	Molnar.	Airplane	Parrot	Lena	Barbara	Boat
BM3D	0.8968	0.90878	0.9348	0.9020	0.8966	0.8951	0.9217	0.8530
WNNM	0.9002	0.9115	0.9406	0.9049	0.8993	0.8969	0.9260	0.8551
EPLL	0.9047	0.9084	0.9362	0.9065	0.9042	0.8894	0.9034	0.8511
DnCNN-S	0.9131	0.9120	0.9501	0.9077	0.9049	0.9003	0.9200	0.8612
DnCNN-B	0.9029	0.9130	0.9462	0.9056	0.8995	0.8999	0.9165	0.8586
FFDNet	0.9118	0.9112	0.949 1	0.9074	0.9045	0.9012	0.9198	0.8608
Ours	**0.9161**	**0.9148**	**0.9524**	**0.9120**	**0.9159**	**0.9038**	**0.9228**	**0.8695**

The bold one in the table is the best indicator.

**Table 9:** SSIM values for different denoising methods on Set12 (δ=25).

Method	C.man	Pepper	Molnar.	Airplane	Parrot	Lena	Barbara	Boat
BM3D	0.8474	0.8696	0.8979	0.8589	0.8528	0.8606	0.8856	0.7998
WNNM	0.8542	0.8746	0.9067	0.8650	0.8553	0.8651	0.8955	0.8014
MLP	0.8593	0.8756	0.9027	0.8682	0.8599	0.8655	0.8616	0.8030
EPLL	0.8460	0.8683	0.8984	0.8636	0.8556	0.8504	0.8427	0.7964
DnCNN-S	0.8407	0.8750	0.9165	0.8540	0.8349	0.8550	0.8540	0.7763
DnCNN-B	0.8645	0.8816	0.9157	**0.8704**	0.8576	0.8690	0.8753	0.8102
FFDNet	0.8755	0.8794	**0.9205**	0.8700	0.8624	0.8736	0.8802	0.8124
OURS	**0.8758**	**0.8862**	0.9201	0.8702	**0.8617**	**0.8746**	**0.8834s**	**0.8179**

The bold one in the table is the best indicator.

**Table 10:** The average value of PSNR (dB) and SSIM of different algorithms on the dataset on Set5 and CBSD68.

Dataset	σ	CBM3D	DnCNN-C	FFDNet	Ours
Set5	15	33.41	34.04	34.30	**34.42**
	25	30.92	31.91	32.10	**32.37**
	50	28.16	28.96	29.25	**29.49**
CBSD68	15	33.50	33.88	33.87	**34.12**
	25	30.69	31.23	31.21	**31.43**
	50	27.36	27.92	27.95	**28.14**

The bold one in the table is the best indicator.

**Table 11:** Average PSNR and SSIM of different methods on two real noise image sets.

	SIDD		DND	
	PSNR (dB)	SSIM	PSNR (dB)	SSIM
CBM3D	25.65	0.685	34.51	0.851
DnCNN-C	35.59	0.861	37.90	0.943
FFDNet	38.27	0.948	37.61	0.942
CBDNet	33.28	0.868	38.06	0.942
Ours	**38.57**	**0.951**	**38.69**	**0.947**

The bold one in the table is the best indicator.

**Table 12:** Comparison of ablation experiment results (δ=25).

Network	BL	BL + NAM	BL + SE	BL + NAM + Direct	BL + NAM + MFM
PSNR	30.45	30.52	30.49	30.54	**30.58**
SSIM	0.8641	0.8652	0.8646	0.8654	**0.8668**

The bold one in the table is the best indicator.

**Table 13:** Comparison of ablation experiment results (δ=50).

Network	BL	BL + NAM	BL + SE	BL + NAM + Direct	BL + NAM + MFM
PSNR	27.35	27.46	27.41	27.48	**27.55**
SSIM	0.7911	0.7931	0.7919	0.7942	**0.7953**

The bold one in the table is the best indicator.

## Data Availability

Data are contained within the article.
